# Identification of adenylate cyclase 2 methylation in bladder cancer with implications for prognosis and immunosuppressive microenvironment

**DOI:** 10.3389/fonc.2022.1025195

**Published:** 2022-10-14

**Authors:** Jianfeng Yang, Jin Xu, Qian Gao, Fan Wu, Wei Han, Chao Yu, Youyang Shi, Yunhua Qiu, Yuanbiao Chen, Xiqiu Zhou

**Affiliations:** ^1^ Department of Surgery, Shangnan Branch of Longhua Hospital Affiliated to Shanghai University of Traditional Chinese Medicine, Shanghai, China; ^2^ Institute of Regenerative Biology and Medicine, Helmholtz Zentrum München, Munich, Germany; ^3^ Wound Treatment Center Affiliated Xinhua Hospital of Medicine College of Shanghai Jiaotong University, Shanghai, China; ^4^ Department of Urology, Renji Hospital, School of Medicine, Shanghai Jiao Tong University, Shanghai, China; ^5^ Longhua Hospital Affiliated to Shanghai University of Traditional Chinese Medicine, Shanghai, China; ^6^ Affiliated Hospital of Youjiang Medical University for Nationalities, Baise, China

**Keywords:** ADCY2, bladder cancer, tumor microenvironment, DNA methylation, prognosis

## Abstract

**Background:**

The incidence and mortality of bladder cancer (BCa) are increasing, while the existing diagnostic methods have limitations. Therefore, for early detection and response prediction, it is crucial to improve the prognosis and treatment strategies. However, with existing diagnostic methods, detecting BCa in the early stage is challenging. Hence, novel biomarkers are urgently needed to improve early diagnosis and treatment efficiency.

**Methods:**

The gene expression profile and gene methylation profile dataset were downloaded from the Gene Expression Omnibus (GEO) database. Differentially expressed genes (DEGs), differentially methylated genes (DMGs), and methylation-regulated differentially expressed genes (MeDEGs) were gradually identified. A cancer genome map was obtained using online gene expression profile interaction analysis, and survival implications were produced using Kaplan-Meier survival analysis. GSEA was employed to predict the marker pathways where DEGs were significantly involved. The study used bisulfite PCR amplification combined with bisulfite amplicon sequencing (BSAS) to screen for methylation analysis of multiple candidate regions of the adenylate cyclase 2 (ADCY2) based on the sequence design of specific gene regions and CpG islands.

**Results:**

In this study, DEGs and DMGs with significantly up- or down-regulated expression were selected. The intersection method was used to screen the MeDEGs. The interaction network group in STRING was then visualized using Cytoscape, and the PPI network was constructed to identify the key genes. The key genes were then analyzed using functional enrichment. To compare the relationship between key genes and the prognosis of BCa patients, we further investigated ADCY2 and found that ADCY2 can be a potential clinical biomarker in BCa prognosis and immunotherapy response prediction. In human BCa 5637 and MGH1 cells, we developed and verified the effectiveness of ADCY2 primers using BSAS technology. The findings revealed that the expression of ADCY2 is highly regulated by the methylation of the promoter regions.

**Conclusion:**

This study revealed that increased expression of ADCY2 was significantly correlated with increased tumor heterogeneity, predicting worse survival and immunotherapy response in BCa patients.

## Introduction

Bladder cancer (BCa) is the most common malignant tumor of the urinary system, and BCa ranks 13th in the incidence spectrum of malignant tumors in China (1), and holds first place in the incidence of urogenital tumors. BCa ranks 9th in incidence and 13^th^ in mortality among all malignant tumors worldwide (2). The etiology of BCa is complex and can occur at any age, and its incidence increases with age. It is a type of malignant tumor that is affected by internal and external influences, with smoking and occupational exposure being the two most obvious pathogenic factors. Current treatments for aggressive BCa include surgery, radiotherapy, and chemotherapy. Chemotherapy is still the primary treatment option in the late stage which includes gemcitabine, cisplatin, carboplatin, paclitaxel, and others (3). However, immunotherapy, targeted therapy, and antibody-coupled drugs are gradually used for the treatment (4, 5), which helps improve the survival rate of patients (6). The most recent clinical studies comprehensively cover all stages of BCa, including the use of new generations of antibody-coupled drugs, targeted drugs, oncolytic viruses, immunomab, dual antibodies, and others (7, 8). The diagnosis and treatment model of early diagnosis, refined surgery, comprehensive multidisciplinary process, and internationalization of clinical translational research were proposed, which significantly improved the diagnosis and treatment of BCa in China (4).

Epigenetics refers to changes in the expression of genes; though environmental factors can cause an organism’s genes to be expressed differently, the genes will not be changed (9). Epigenetics processes include DNA methylation (10), genomic imprinting, maternal effects, gene silencing, dormant transposon activation, and RNA editing. Among these, DNA methylation refers to the covalent binding of a methyl group to the 5’carbon site of the CpG dinucleotide in the genome under the action of DNA methylating transferase (11, 12). It can control gene expression by causing changes in chromatin structure, DNA conformation, DNA stability, and interaction of DNA with proteins (13). DNA methylation is not a permanent change; it is reversible. Therefore, DNA methylation and demethylation modification have a wide range of applications and is associated with genetic imprinting and cancer (14, 15). Aberrant methylation can even turn normal stem cells into cancer stem cells, a sign of cancer development and progression. The researchers found that cancer cell genomes are characterized by methylation or alternative splicing events by examining methylation patterns on DNA in healthy human organs and malignant tissues (16, 17). For example, the obesity-associated protein (FTO) has been found to be overexpressed in BCa, which stimulates cancer cell metabolism and subsequently causes tumorigenesis and progression (18).

Currently, a machine learning model for predicting immunotherapy response based on tumor DNA methylation characteristics has been developed exploratively (19, 20). Methylation and genomic features are anticipated to develop into a potential research direction for tumor immune microenvironment and tumor immunotherapy marker screening using the selected methylation feature set to predict the response of pan-cancer species to immunotherapy (21–23). However, the limitation of tumor immunotherapy is that some cancer patients may not respond to such drugs and are prone to severe immune-related adverse events (irAEs), which can lead to various local and systemic autoimmunity. DNA methylation is another biomarker that is expected to be used as a predictor of immunotherapy efficacy (24, 25). In addition to its role in tumorigenesis by regulating gene expression and promoting somatic and structural mutations, DNA methylation can assess the status of the tumor immune microenvironment. Previous studies have shown that DNA methylation characteristics can effectively predict the proportion of different types of immune cells in the tumor microenvironment, and the methylation level is related to the efficacy of immunotherapy (26). Many clinical trials of BCa immunotherapy are underway, but no efficacy has been positive to date. Studies have shown that CDH7 and LUZP1 are associated with the clinical characteristics of BCa, but more biomarkers for predictive immunotherapy and new effective therapeutic targets are still needed (27).

The current diagnosis and treatment technology has not kept up with the level of research due to the high incidence and relapse of BCa, and there are currently no reliable biomarkers for immunotherapy. Therefore, we are searching for reliable markers for early diagnosis and treatment of BCa patients to improve the survival rate and quality of life.

## Methods

### Acquisition and standardization of raw microarray dataset

We downloaded the gene expression profiling dataset created by high-throughput sequencing (GSE37815) and the microarray-based gene methylation profiling dataset (GSE37817) from the Gene Expression Omnibus database (GEO, https://www.ncbi.nlm.nih.gov/geo/). In total, five normal bladder mucosae and 18 primary BCa samples were included in GSE37815 (platform: GPL6102 Illumina human-6 v2.0 expression beadchip). As for the DNA methylation datasets, GSE37817 included six normal bladder mucosae and 18 primary BCa samples based on the GPL8490 platform (Illumina HumanMethylation27 BeadChip).

### Identification of methylation-regulated differentially expressed genes

To identify the potential prognostic hub genes of the MeDEGs, we performed GEO2R (http://www.ncbi.nlm.nih.gov/geo/geo2r/) to compare two or more groups of samples in a GEO Series to screen genes that are differentially expressed across specific experimental conditions. In the present study, GEO2R was used to identify the differentially expressed genes (DEGs) and differentially methylated genes (DMGs). |t| >2 and P <0.05 were considered statistically significant. Furthermore, hypomethylation-high expression genes were obtained after the overlap of upregulated and hypomethylated genes, and hypermethylation-low expression genes were obtained after the overlap of downregulated and hypermethylated genes. The hypomethylation-high expression genes and hypermethylation-low expression genes were then identified as methylation-regulated differentially expressed genes (MeDEGs).

### Functional enrichment analysis

To obtain the functional annotations of hub gens, we utilized the Database for Annotation, Visualization, and Integrated Discovery (DAVID, https://david.ncifcrf.gov/) is a straightforward web tool that can provide integrative and systematic annotation for users to unravel the biological interactions of multiple genes. It was utilized to perform functional and pathway enrichment analyses. Gene ontology (GO) analysis, including the biological process (BP), cellular component (CC), molecular function (MF), and Kyoto Encyclopedia of Genes and Genomes (KEGG) pathway enrichment analysis, were conducted for the selected MeDEGs by DAVID (28, 29). *P <*0.05 was considered statistically significant.

### PPI network construction and identification of hub genes

In this study, STRING (http://string-db.org; version 11.0) was adopted to describe protein co-regulation of hypomethylation-high expression genes and hypermethylation-low expression genes, respectively, and measure functional interactions among nodes (30). The interaction specificity score above 0.4 (the default threshold in the STRING database) was considered statistically significant. Cytoscape (version 3.6.0) was used to visualize interaction networks obtained from STRING (31). MCODE (version 1.4.2) of Cytoscape is a plug-in to cluster a given network to identify densely connected regions based on topology (32). It was utilized to find the most related module network with selection threshold as follows: MCODE scores >5, degree cutoff = 2, node score cut-off = 0.2, Max depth = 100 and k-score = 2.

### Survival and hierarchical analysis

Gene Expression Profiling Interactive Analysis (GEPIA, http://gepia.cancer-pku.cn/) is an online tool that can provide customizable functionalities based on data from The Cancer Genome Atlas (TCGA; https://tcga-data.nci.nih.gov/tcga/) and the Genotype-Tissue Expression project (GTEx; https://www.gtexportal.org/home/index.html) (33). GEPIA performs survival analysis based on gene expression levels, using a log-rank test for the hypothesis evaluation. The horizontal axis (x-axis) represented the time in days, and the vertical axis (y-axis) showed the probability of surviving or the proportion of people surviving. The cut-off value was defined *via* median value or using “survminer” R package. The lines presented the survival curves of the two groups.

### Data processing of gene set enrichment analysis

Based on data from the TCGA database, the GSEA tool (version 2.10.1 package) was used to predict associated up- and down-regulated genes and their significantly involved hallmarks pathways (34). The student’s t-test statistical score was implemented in consistent pathways, and the mean of the DEGs was calculated for each analysis. A permutation test 1000 times was utilized to recognize the significantly involved pathways. The adjusted P using Benjamini and Hochberg (BH) and false discovery rate (FDR) method by default were used to correct for the occurrence of false positive results. Significantly related genes were defined with an adjusted P <0.01 and FDR <0.25.

### Bisulfite PCR amplification and bisulfite amplicon sequencing technology

BSAS methylation next-generation sequencing of BCa cell lines was conducted by GeneChem Biotechnology Co., Ltd., Shanghai (GSGC0257632). Microsoft Office Excel software and Methylation Plotter software were used to examine the results. The Kruskal-Wallis test is a nonparametric test of three or more groups of data and was used when the ANOVA test could not be utilized. Bisulfite PCR amplification was performed using the High Pure PCR Template Preparation Kit (Roche) with forward and reverse primers provided. DNA methylation libraries were developed using the VAHTS Turbo DNA Library Prep Kit for Illumina^®^ (ND102-0102).

## Results

**Figure 1:**
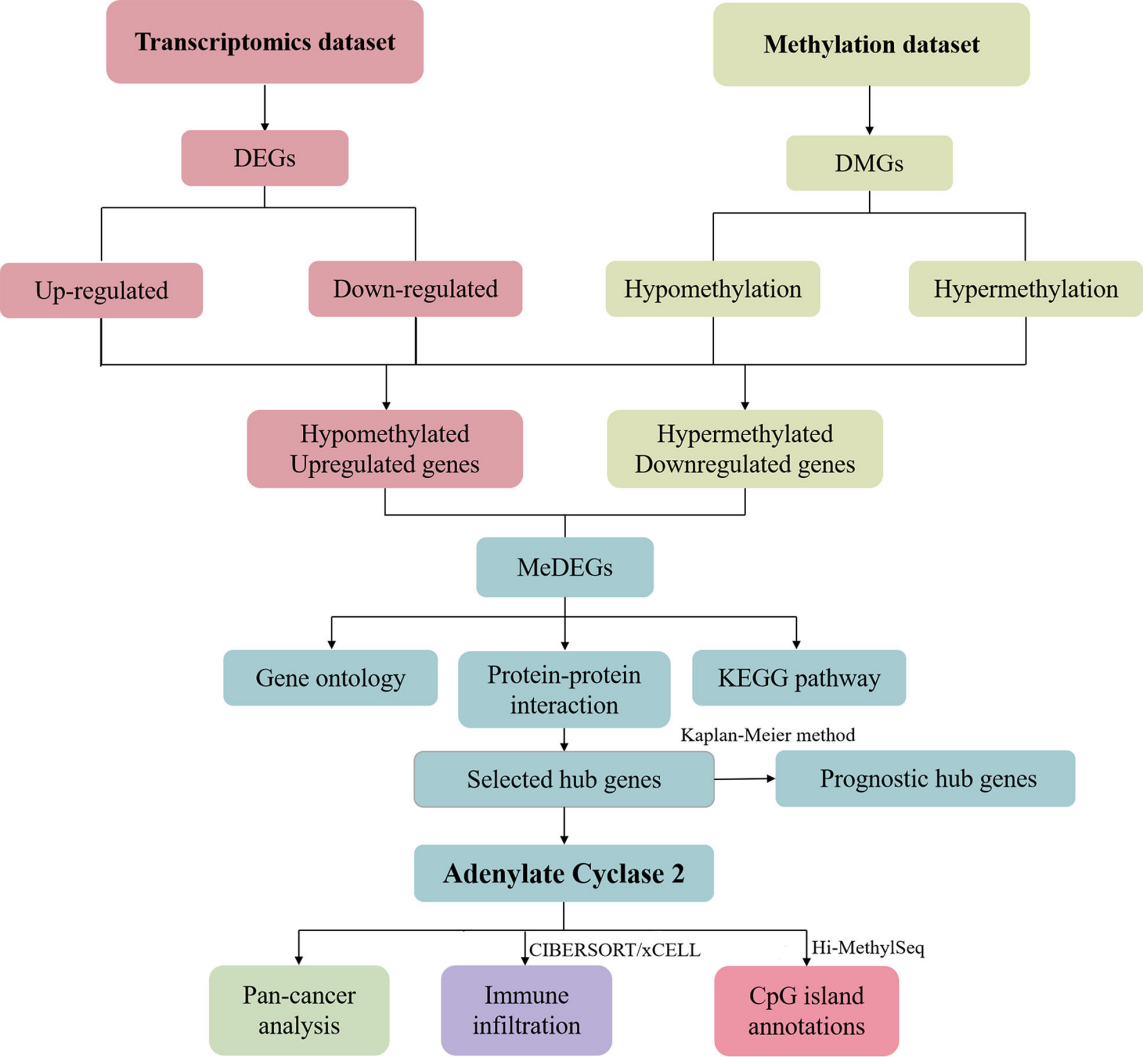
Schematic diagram of the study.

We selected DEGs and DMGs based on the transcriptome dataset and methylation dataset. The up-or down-regulated genes were selected to find the intersection, and the methylation-related differential genes were eliminated. We identified the key gene ADCY2 through a series of mRNA and protein level analyses of these genes, including functional enrichment analysis and protein interaction network. Further, the survival, immune infiltration, and CpG island location analyses were carried out around ADCY2 in the schematic diagram **(** **Figure 1** **)**.

### Identification of MeDEGs in BLCA

**Figure 2:**
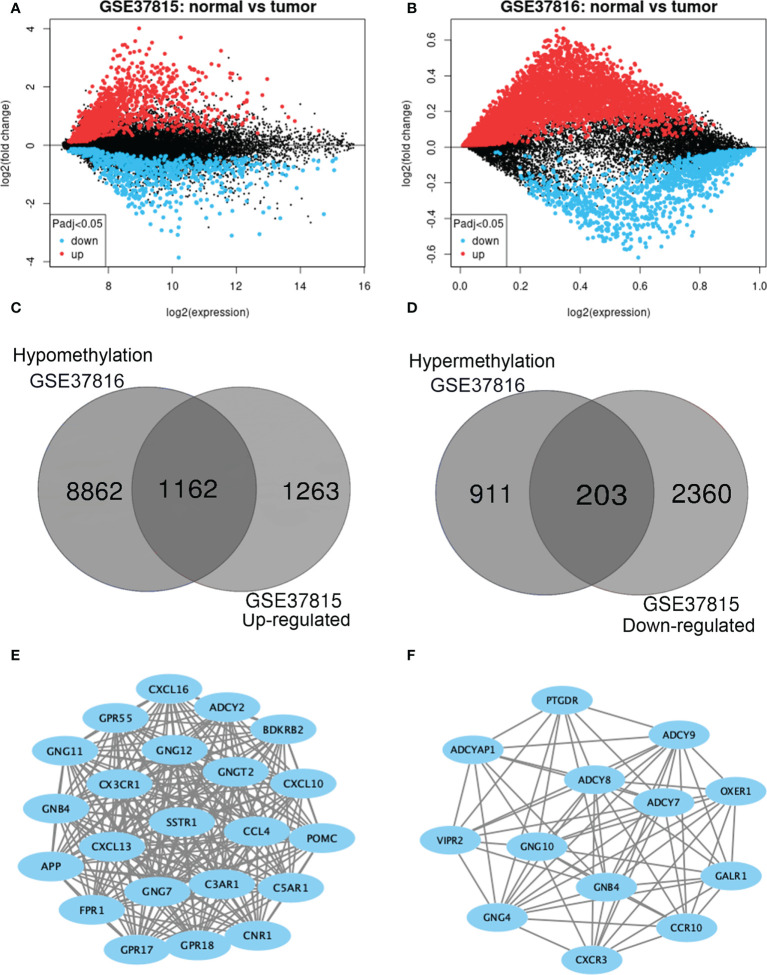
Identification of methylation-regulated differentially expressed genes (MeDEGs). **(A, B)** Mean difference plot of significantly differentially expressed genes (DEGs) in two independent validation sets. Red represents up-regulated genes, and blue represent down-regulated genes. **(C, D)** The up-regulated and down-regulated genes in the two validation sets were selected as intersections, respectively. **(E, F)** Constructed protein-protein interaction networks based on common genes.

GEO2R was adopted to identify the DEGs and DMGs, respectively. For DEGs of gene expression microarray, 2425 overlapping up-regulated genes and 2563 overlapping down-regulated genes were screened **(** **Figures 2A, B** **)**. A total of 1114 overlapping hypermethylation genes and 10,024 overlapping hypomethylation genes were discovered for DMGs of gene methylation microarray. The study identified 1162 hypomethylated, upregulated genes and 203 hypermethylated, downregulated genes after integrating the DEGs and DMGs (**Figures 2C, D**).

### PPI network establishment and hub genes

The PPI network of hypomethylation-upregulated genes and hypermethylation-downregulated genes was visualized using Cytoscape (version 3.6.0) [28]. A Cytoscape plug-in called MCODE (version 1.4.2) clusters a given network to select densely connected regions based on topology [29]. The results are presented in **Figures 2E, F**. As a result, in the hypomethylation-upregulated genes module, CXCL10, CXCL16, CX3CR1, SSTR1, C3AR1, CNR1, ADCY2, BDKRB2, POMC, GPR55, GNG12, GNG11, GNGT2, CCL4, C5AR1, GNB4, CXCL13, GNG7, GPR18, APP, FPR1, GPR17, and GPR18 were confirmed as hub genes. While in the hypermethylation-downregulated genes module, PTGDR, ADCY9, OXER1, GALR1, ADCYAP1, ADCY8, ADCY7, VIPR2, GNG10, GNB4, CCR10, GNG4, and CXCR3 were confirmed as hub genes.

### Functional enrichment analysis of MeDEGs

For hypomethylation-upregulated genes, changes in biological processes were mostly enriched in angiogenesis, signal transduction, aging, and immune response. The hypermethylation-downregulated genes were primarily enriched in extracellular matrix organization, signal transduction, cell adhesion, cAMP-mediated signaling, and cellular response to glucagon stimulus. Moreover, the study found that the hypomethylated, upregulated genes were associated with extracellular exosome, plasma membrane, and extracellular region. Whereas the hypermethylated, downregulated genes were associated with the proteinaceous extracellular matrix, plasma membrane, and extracellular matrix in the cellular component group. For hypomethylated, upregulated genes, changes in molecular function were primarily enriched in protein binding, heparin-binding, actin filament binding, and extracellular matrix structural constituent. On the other hand, for hypermethylated, downregulated genes, changes were significantly enriched in collagen binding, phosphorus-oxygen lyase activity, and extracellular matrix binding. Furthermore, pathway enrichment was performed using KEGG. The study revealed that hypomethylated genes predominantly participated in morphine addiction, retrograde endocannabinoid signaling, and cholinergic synapse. For hypermethylated genes, the most significantly enriched pathways involved focal adhesion, pathways in cancer, and the PI3K-Akt signaling pathway.

### Survival analysis

**Figure 3:**
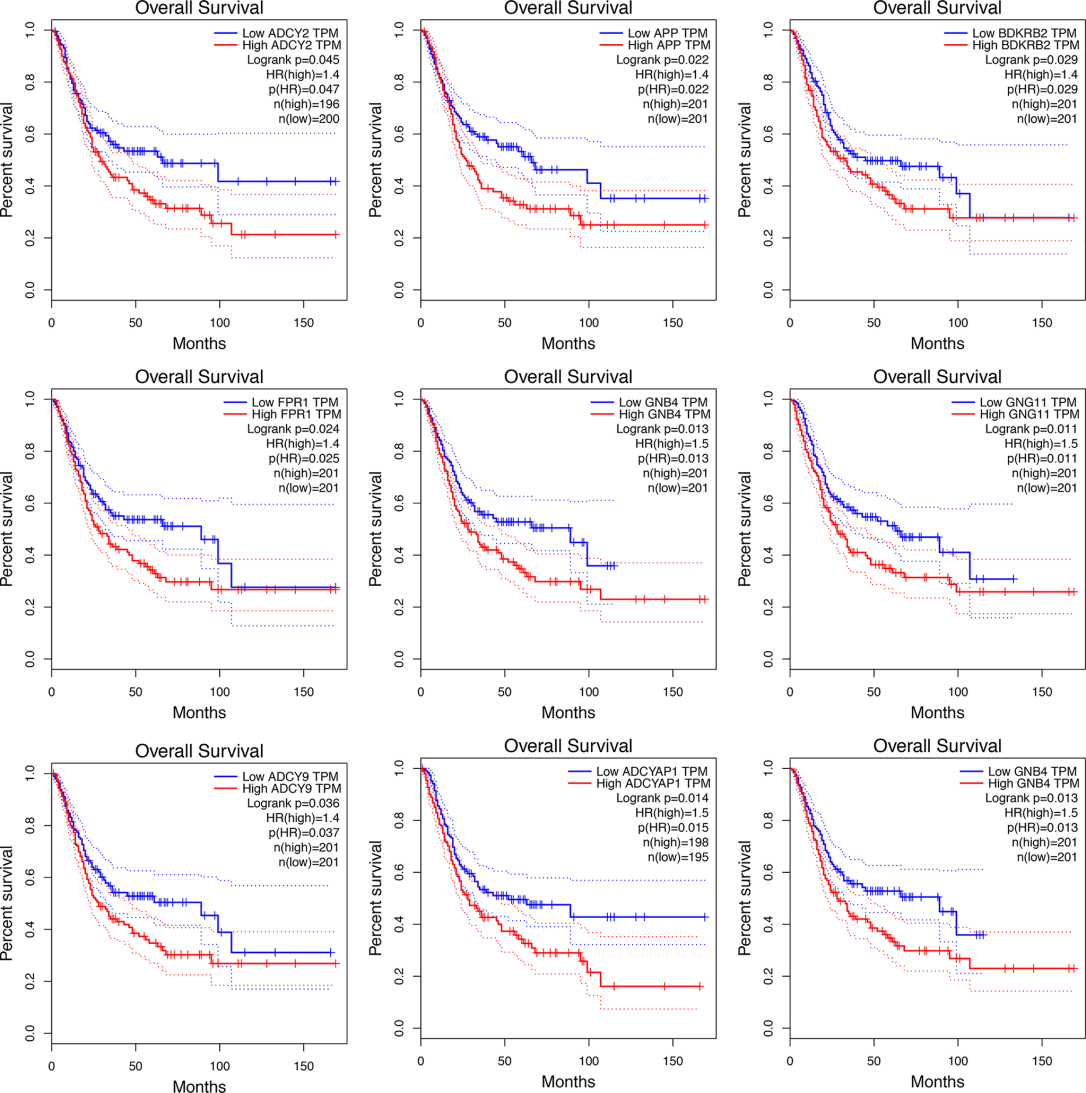
The KM curve indicates the overall survival of the selected differential genes.

Significant survival outcomes of hub genes in the PPI network are displayed in **Figure 3**. According to the expression of each gene, overall survival for SKCM patients was acquired. The study found that high mRNA expression of ADCY2 (*P* = 0.047) was significantly associated with worse prognosis for SKCM as well as APP (*P* = 0.022), BDKRB2 (*P* = 0.029), FPR1 (*P* = 0.025), GNB4 (*P* = 0.013), GNG11 (*P* = 0.011), ADCY9 (*P* = 0.036), and ADCYAP1 (*P* = 0.014). Significant genes and pathways were obtained using GSEA.

### Differential expressed genes

**Figure 4:**
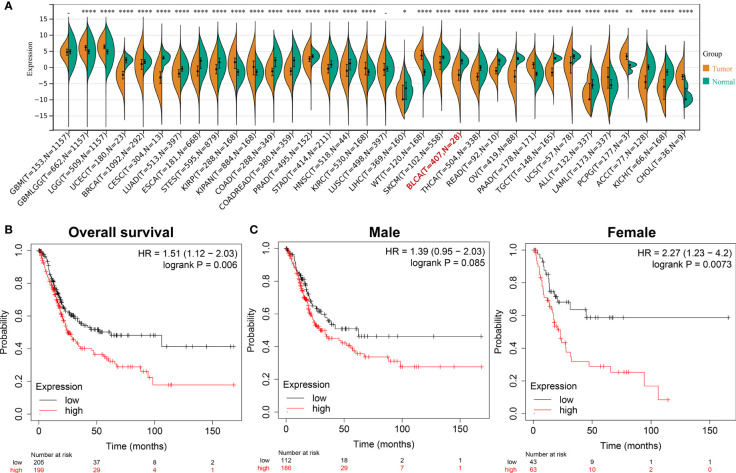
Survival outcomes of ADCY2 expression in cancers and novel role in BCa. **(A)** Differential expression of ADCY2 in tumor and normal tissues in pan-cancer. **(B)** Overall survival curve of BCa patients. **(C)** Survival curves of male and female in high and low expression groups. *p<0.05; **p<0.01; ****p<0.0001.

After screening and analysis, this study found that ADCY2 was significantly associated with prognosis. A total of 34 cancers were then selected to observe the difference in ADCY2 expression between tumor and normal tissues. In BCa, the expression level in tumor tissues was significantly lower than that in normal tissues (n = 435, **Figure 4A**). Hence, a separate survival analysis of BCa patients and male and female subgroups was performed. The findings revealed that the high expression of ADCY2 predicted a worse prognosis, and it was significant in female patients, which might be due to a higher incidence of BCa in males than in females **(** **Figures 4B, C** **)**.

**Figure 5:**
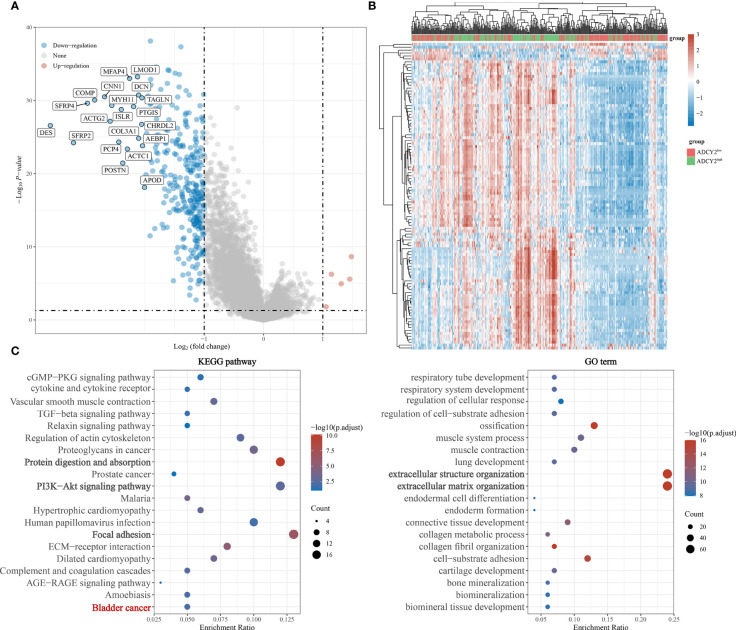
Functional enrichment annotations of ADCY2 expression in BCa. **(A)** A volcano plot of differentially expressed genes (DEGs), with down-regulated genes in blue and up-regulated genes in red. **(B)** Heat map of ADCY2 and DEGs. The low ADCY2 expression groups in red (at the top) and the high ADCY2 expression groups in green. Red and blue represent expression trends corresponding to up-regulated and down-regulated genes. **(C)** KEGG pathway and GO term functional enrichment analysis.

Simultaneously, the significantly down-regulated genes were listed, including MFAP4, LMOD1, CNN1, COMP, SFRP4, and so on **(** **Figure 5A** **)**. The correlation trend of these genes in populations with high and low ADCY2 expression was observed **(** **Figure 5B** **)**.

### Functional enrichment analysis

In the KEGG pathways, ADCY2 was found to be primarily enriched with protein absorption, PI3K-Akt signaling pathway, and focal adhesion pathway. An enrichment of ADCY2 was also observed in the BCa pathway, which showed that ADCY2 is involved in the development of BCa. Meanwhile, in GO term, ADCY2 was mainly enriched in extracellular structure organization and extracellular matrix organization pathways **(** **Figure 5C** **)**.

### Immune correlation analysis of ADYC2 expression in BCa

**Figure 6:**
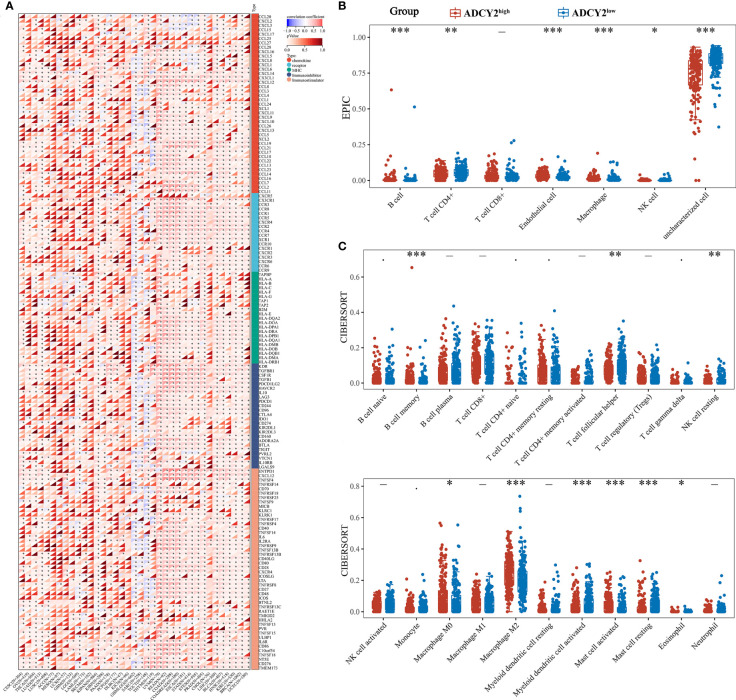
Implications of ADCY2 expression in immune regulators and tumor-infiltrated lymphocytes of cancers. **(A)** Correlation between ADCY2 and several immune-related cytokines in pan-cancer. **(B, C)** Immune infiltration difference analysis between high and low ADCY2 groups with EPIC and CIBERSORT algorithms. *p<0.05; **p<0.01; ***p<0.001.

Several immune-related cytokines were screened, mainly from five families, for correlation analysis with ADCY2 in pan-cancer. ADCY2 was found to be associated with most cytokines and presented a tumor immune microenvironment dominated by MHC and chemokine in most cancer types **(** **Figure 6A** **)**. Notably, an immune infiltration analysis was performed in BCa using EPIC and CIBERSORT algorithms. T cell CD4+ memory resting, B cell naive, B cell memory, and macrophage M2 were significantly clustered in the ADCY2^high^ group, while uncharacterized cells were significantly increased in the ADCY2^low^ group **(** **Figures 6B, C** **)**.

**Figure 7:**
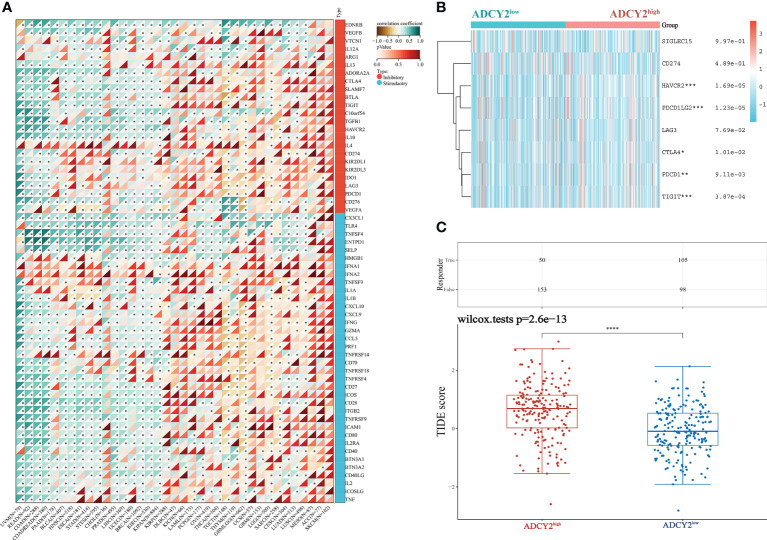
Relationship between ADCY2 expression and immune checkpoints in cancers and its implications in immunogenicity and tumor heterogeneity of BCa. **(A)** Molecular correlation analysis of ADCY2 and immune checkpoint in pan-cancer. **(B)** Heat map association between ADCY2 high and low groups and critical immune checkpoints in BCa. **(C)** TIDE scores in two groups of BCa samples. *p<0.05; **p<0.01; ***p<0.001; ****p<0.0001.

Next, the study screened for immune checkpoint molecules. The association between ADCY2 and some immune checkpoints in pan-cancer was first explored, including those that promote immunotherapy and inhibit the efficacy of immunotherapy **(** **Figure 7A** **)**. The immune molecules, such as HAVCR2, PDCD1LG2, and TIGIT, which were significantly clustered in the high and low ADCY2 groups of BCa, were then studied independently (**Figure 7B**). However, the difference between the two groups was not significant. Finally, TIDE analysis was performed in the two BCa sample groups. The high score of the ADCY2^high^ group suggested a high expression of ADCY2 associated with tumor heterogeneity, indicating a worse immunotherapy effect and prognosis for BCa patients **(** **Figure 7C** **)**.

### Identification of promoter regions of ADCY2 methylation in BCa cells

**Figure 8:**
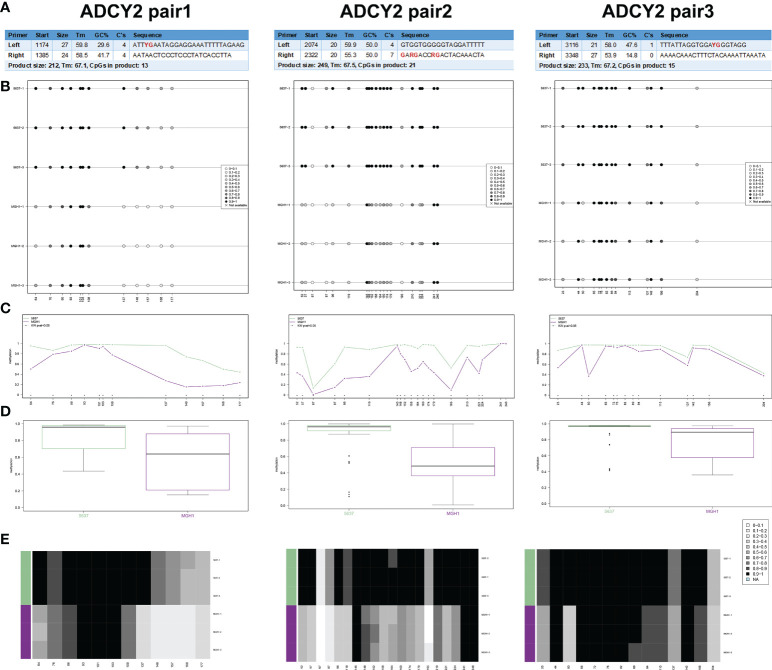
Identification of promoter regions of ADCY2 methylation in BCa cells using BSAS technology. **(A)** The effectiveness of ADCY2 primers was developed and verified in human BCa 5637 and MGH1 cells using BSAS technology. The three pairs of primers for ADCY2 methylation were shown, and the CG site of the amplified fragment was indicated in bold and red. **(B)** The schematic diagram of the average methylation information of all sites in the 5637 and MGH1 cells. **(C–E)** The methylation levels of all sites in the grouped samples were shown in boxplots and dendrograms.

In human BCa 5637 and MGH1 cells, the study developed and verified the effectiveness of ADCY2 primers using BSAS technology. **Figure 8A** displays the three pairs of primers for ADCY2 methylation, where the CG site of the amplified fragment is indicated in bold and red. The schematic diagram of the average methylation information of all sites in the 5637 and MGH1 cells was developed using the Methylation plotter (**Figure 8B**). Besides, the methylation levels of all sites in the grouped samples are shown in boxplots and dendrograms (**Figures 8C–E**). The findings revealed that the expression of ADCY2 is highly regulated by the methylation of the promoter regions.

## Discussion

BCa is a common malignant tumor of the urinary system. The incidence of BCa ranks 9th among all malignant tumors and 7th among male malignant tumors worldwide (6). Currently, surgery is the primary treatment method, assisted by chemotherapy and radiotherapy (5). However, many patients disqualify for radical cystectomy or refuse the therapy. Cancer immunotherapy, which harnesses the immune system of individuals to fight cancer, has revolutionized cancer treatment strategies. However, the majority of patients show no clinical response, and the mechanisms of resistance remain unclear (35–37). Therefore, there is an urgent need for novel immunotherapies and therapeutic targets.

In this study, the DEGs and DMGs from the transcriptomics dataset and methylation dataset were respectively studied. The up-regulated or down-regulated DEGs and DMGs were selected. The methylation-related differential gene sets were screened by the method of intersection using the GEO2R website to identify the DEGs as well as the DMGs. Then Cytoscape (version 3.6.0) was used to visualize the interaction networks group from STRING, based on which a PPI network was created, and the key genes were obtained. The functional enrichment analysis of key genes was carried out. For hypomethylation-upregulated genes, changes in biological processes were mainly enriched in angiogenesis, signal transduction, aging, and immune response. The hypermethylation-downregulated genes were primarily enriched in extracellular matrix organization, signal transduction, cell adhesion, cAMP-mediated signaling, and cellular response to glucagon stimulus. To compare the relationship between key genes and the prognosis of BCa patients, we further studied ADCY2, which can significantly predict the prognosis.

After a pan-cancer comparative study, immunoinfiltration analysis, real-world cohort validation, and CpG island annotations, we found that patients with high ADCY2 expression had significantly shorter overall survival and less effective immunotherapy. However, in the pan-cancer analysis, we found that the expression level of ADCY2 in tumor tissues was lower than that in normal tissues, which may be due to hypermethylation. Due to the significant immune escape of BCa cells, many immunotherapies are ineffective for all patients and are accompanied by immune rejection and side effects (38). Currently, atezolizumab (Tecentriq), pembrolizumab (Keytruda), nivolumab (Opdivo) (39), and others are approved for the treatment of locally progressive and metastatic BCa that has failed platinum-based chemotherapy (40). Both atezolizumab and pembrolizumab are also approved by the FDA for first-line treatment of patients who are not eligible for platinum-based chemotherapy (41). However, some patients do not respond to these drugs, and only 20 percent benefit from them (42).

This study found that the ADCY2 gene can be used as a biological indicator for the diagnosis and immunotherapy of BCa patients. Moreover, increased ADCY2 expression is associated with worse prognosis, higher tumor heterogeneity, and worse immunotherapy effect. Though ADCY2 has been found to be a novel lipid prognostic feature in head and neck squamous cell carcinoma (43), it has not been studied in BCa. The relevant conclusions of this study innovatively discovered the differential expression of ADCY2 in BCa and proved that the expression of ADCY2 is highly regulated by the methylation of the promoter regions and could be used as a reliable biomarker in the diagnosis and treatment of BCa patients.

## Data availability statement

The original contributions presented in the study are included in the article/**Supplementary Material**. Further inquiries can be directed to the corresponding author.

## Author contributions

Conceptualization: JY, JX, YS, YQ and FW. Data curation and formal analysis: JY, WH, CY, XZ, and FW. Investigation and methodology: JY, JX, QG, WH and FW. Resources and software: JY, JX, QG, CY, XZ and YC. Supervision: XZ, YQ and YC. Validation and visualization: QG, FW and YC. Original draft: JY. Editing: QG, XZ, YQ and YC. All authors contributed to the article and approved the submitted version.

## Acknowledgments

We are grateful to all patients for their dedicated participation in the current study. We thank Bullet Edits Limited for the linguistic editing and proofreading of the manuscript.

## Conflict of interest

The authors declare that the research was conducted in the absence of any commercial or financial relationships that could be construed as a potential conflict of interest.

## Publisher’s note

All claims expressed in this article are solely those of the authors and do not necessarily represent those of their affiliated organizations, or those of the publisher, the editors and the reviewers. Any product that may be evaluated in this article, or claim that may be made by its manufacturer, is not guaranteed or endorsed by the publisher.
